# The probability of detecting host-specific microbial source tracking markers in surface waters was strongly associated with method and season

**DOI:** 10.1128/spectrum.01972-24

**Published:** 2024-12-17

**Authors:** Claire M. Murphy, Daniel L. Weller, Tanzy M. T. Love, Michelle D. Danyluk, Laura K. Strawn

**Affiliations:** 1 School of Food Science, Washington State University Irrigated Agriculture Research and Extension Center, Prosser, Washington, USA; 2 Department of Food Science and Technology, Virginia Tech, Blacksburg, Virginia, USA; 3 Department of Biostatistics and Computational Biology, University of Rochester Medical Center, Rochester, New York, USA; 4 Department of Food Science and Human Nutrition, Citrus Research and Education Center, University of Florida, Lake Alfred, Florida, USA; The University of Tennessee Knoxville Food Science, Knoxville, Tennessee, USA

**Keywords:** microbial source tracking, fecal contamination, surface water, methods

## Abstract

Fecal contamination of surface waters presents significant human health and environmental risks. While many methods for understanding fecal contamination cannot differentiate between human and animal sources, microbial source tracking (MST) marker methods are used to identify fecal sources. To understand how to best employ MST marker data for managing fecal contamination risks, the present study compiled previously collected MST marker data (*N* = 12,878 samples) from across North America; each sample in the data set had been tested for fecal contamination from one or more of five sources (avian, canine, human, ruminant, swine). Using these data, this study aimed to characterize associations between non-methodological and methodological factors and detection of host-specific MST markers and determine how methodological differences may complicate the interpretation of these associations between studies. Variance partitioning analysis was used to quantify the variance in host-specific MST marker detection attributable to non-methodological and methodological factors. Conditional forest and regression analysis were utilized to assess the association between detection and select non-methodological and methodological factors. Between 10% (canine) and 38% (human) of total variance was uniquely attributable to non-methodological factors for any of the fecal sources considered, while between 50% (human) and 84% (canine) of variance could not be attributed to either methodological or non-methodological factors. This highlights the need for standardization of methods for MST marker detection across studies but also suggests that other factors, beyond those non-methodological variables considered here, influenced variation in the likelihood of MST marker detection.

**IMPORTANCE:**

This study underscores complications associated with comparing findings from studies that used different methodologies to detect the same fecal targets and highlights the difficulties associated with using non-comparable data to generalize findings and develop science-based risk management plans. These findings highlight the need for standardization of sampling and laboratory methods across microbial source tracking marker studies. Our findings build on previous research to suggest that one-size-fits-all approaches to managing fecal hazards in surface waterways may not be appropriate; instead, strategies tailored to specific water sources and conditions at the time of water use may be more effective.

## INTRODUCTION

Fecal contamination of recreational and agricultural surface water is a public health and environmental concern (1–3). Fecal contamination of surface water can originate from numerous sources including humans (e.g., untreated sewage, septage), domesticated animals (e.g., agricultural runoff from manure), or native wildlife (e.g., waterfowls). Quantification of fecal indicator bacteria (e.g., generic *Escherichia coli*) is used to identify when and where fecal contamination of surface waterways has occurred with elevated levels of fecal indicator bacteria considered indicative of an increased risk of fecal contamination. However, since methods used to quantify fecal indicator bacteria cannot distinguish between different sources of fecal contamination (e.g., human, avian), microbial source tracking (MST) marker methods can be employed to further pinpoint the origins of fecal contamination in waterways. MST marker methods, which target host-associated sequences/markers or microorganisms by molecular-based methods, aim to identify the source of fecal contamination in the environment, particularly in surface water (1, 4–9). *Bacteroidales*, a group of anaerobic bacteria found abundantly in the gastrointestinal tracts of humans and animals, are commonly employed MST markers for identifying fecal contamination sources in water as these bacteria have high host specificity, are abundant in fecal matter, can survive in the environment longer than other indicators, and are widely accepted by regulatory agencies (10).

Many MST marker methods and techniques have been developed to discriminate between sources of fecal contamination, including human (11–17), ruminant (15, 18), avian (19, 20), or other animal sources (21, 22). However, past studies examining MST markers within a single waterway or region scale have found differences in marker abundance recovery due to environmental, spatial, and temporal factors, such as water and air temperature (23, 24), adjacent land usage (25), amount of precipitation (25–27), and season (25, 26). An improved understanding of the variability in marker detection and abundance across various water types, regions, and environmental conditions can provide important insights into the dynamics of host-specific MST markers in waterways. Such knowledge on how these factors impact MST marker recovery is essential for developing effective management strategies to protect water quality and public health, and for determining if management practices need to be tailored to specific conditions.

While numerous studies have surveyed waters for various host-specific MST markers, the sample collection and laboratory processing methods are diverse and often vary by laboratory and available resources. Previous research has found that when different methods are used (e.g., the volume of water sampled, the genetic marker used for confirmation), differences in the results are observed (e.g., prevalence, quantities) (2, 7, 10, 16, 28). A previous study that assessed the sensitivity and specificity of 41 different methods for detecting MST markers by 27 independent laboratories found evidence of considerable differences in results between laboratories and methods (16). These inconsistencies emphasize that diverse protocols and sample processing techniques for MST markers in water hinder the comparability of findings (e.g., prevalences and associations) across studies. This undermines our ability to determine whether environmental differences genuinely drive contamination risks or if the observed variations are due to the methods used.

To address gaps in knowledge, the present study gathered a data set of MST marker results spanning various water types and geographical regions within North America, encompassing diversity in collection, processing, and laboratory techniques. Based on this data set, this study aimed to (i) measure the influence of methodological differences on host-specific fecal contamination and (ii) determine whether differences in water quality due to methods could be differentiated from other pertinent non-methodological factors (e.g., water source, geographic location) in order to assess whether management strategies should be tailored to specific waterway and environmental factors.

## RESULTS

### Distribution of host-specific microbial source tracking markers

Following data compilation and cleaning, 4,906 unique water samples were retained for analysis; the majority of water samples were tested for more than one host-specific MST marker (Table 1). Human (*N* = 4,757), avian (*N* = 2,907), ruminant (*N* = 2,537), canine (*N* = 2,166), and porcine (*N* = 511) MST marker data were collected between 2008 and 2021 from waterways in 14 US states and the District of Columbia (DC). Samples were most frequently collected in summer (*N* = 2,875), followed by spring (*N* = 884), fall (*N* = 789), and winter (*N* = 358). Samples were most frequently collected from streams (*N* = 1,804), the Great Lakes (*N* = 1,518), non-tidal rivers (*N* = 1,259), estuaries/tidal rivers (*N* = 54), groundwater (*N* = 38), and canals (*N* = 11) (Table 1). It is noteworthy that although the distribution of data for each marker varied across water types, samples from the Great Lakes, rivers, and streams constituted the majority of samples tested for each host-specific MST marker.

**TABLE 1:** Summary of the data sets

Data source	Org. type^*a*^	State	Human MST		Ruminant MST		Canine MST		Avian MST		Porcine MST		Reference
			P^*b*^	N^*c*^	P	N	P	N	P	N	P	N	
Boehm Lab, Stanford U	U	CA	15	212	3	238	–	–	–	–	6	181	(29)
Colwell Lab, U of MD	U	DC	5	3	–^*d*^	–	0	4	–	–	–	–	(30)
Food Safety Lab, Cornell U	U	NY	49	147	34	162	1	195	8	188	–	–	(31)
Green Lab, SUNY College of Env Sci and Forestry	U	NY, UT	235	458	297	396	112	580	428	236	–	–	(5)
Harwood Lab, U of South FL	U	FL	0	84	7	77	–	–	–	–	–	–	(27)
McLellan Lab, U of W	U	MI, OH, WI	474	64	102	117	–	–	–	–	–	–	(25, 32)
Onondaga Env Inst	N/C	NY	22	9	11	21	0	32	0	32	–	–	(6)
Public Health Lab, Humboldt Co, CA/U of South FL	G, U	CA	4	197	41	160	13	188	50	151	–	–	(33)
Richardson Lab, Cornell U	U	NJ, NY	38	27	–	–	–	–	86	58	–	–	(34, 35)
Shrestha Lab, U of IL at Chicago	U	IL	10	185	–	–	13	182	45	150	–	–	(36)
US Geological Survey	G	DE, GA, SC, IL, IN, WI	782	1,596	63	667	184	662	883	592	72	252	(26, 37–42)
US Env Prot Agency	G	GA	97	44	107	34	–	–	–	–	–	–	(43)

^*a*^ Organization type: G, governmental organization; N/C, non-profit or citizen science organization; U, university.

^*b*^ Number of samples testing positive.

^*c*^ Number of samples testing negative.

^*d*^ No samples tested.

All studies used molecular methods for detection. The molecular marker(s) used varied widely between studies; moreover, some studies only used one marker to detect feces attributable to a given host (e.g., humans), while other studies used multiple markers for each host. For example, of 4,757 samples tested for human fecal markers, 91% (4,347) were tested for HF183, 15% (728) for Mnif, 5% (260) for HumM2, and 3% (144) for *Bacteroides thetaiotaomicron (B. theta*). Studies also varied in sampling method and volume. While the majority of samples represent grab samples (including all samples tested for avian, canine, and porcine markers), a small number of samples represented flow-weighted grab samples. The flow-weighted grab samples were only tested for human (8%; 399/4,757) and/or ruminant (9%; 219/2,537) markers and were collected from rivers in MI, OH, or WI (Table 1). Sample volume ranged from 20 mL to 1,000 mL. Variation in sample volume was lower for samples tested for avian (interquartile range [IQR] = 443 mL), canine (IQR = 291 mL), and porcine (IQR = 158 mL) markers compared to samples tested for human (IQR = 760 mL) and ruminant (IQR = 604 mL) markers; this may reflect the fact that more samples and laboratories tested for human and ruminant markers compared to avian, canine, and porcine samples.

### Variance in MST marker detection attributable to methodological and non-methodological factors

**Fig 1:**
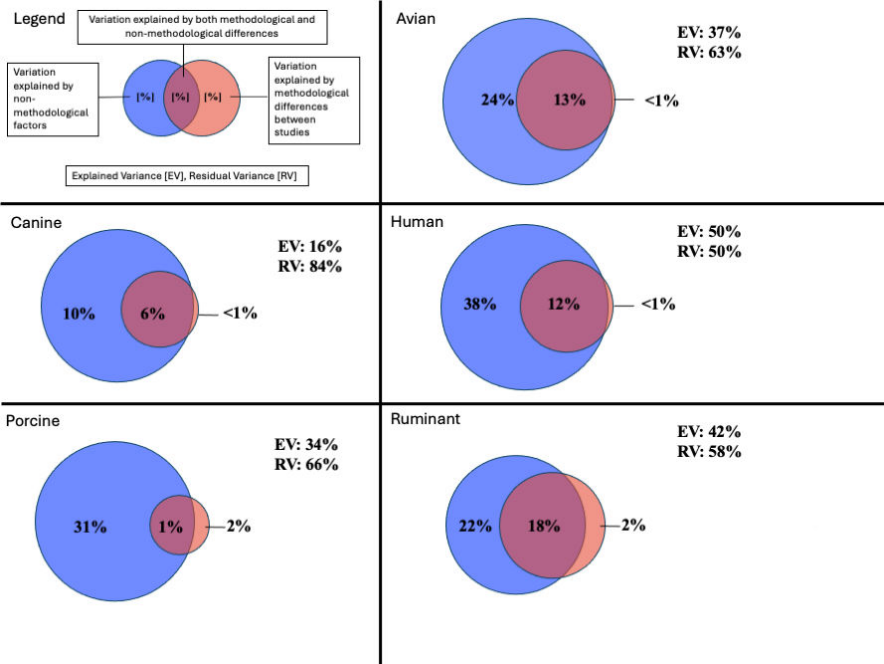
Variance in the likelihood of detecting avian, canine, human, porcine, and ruminant MST markers that is jointly versus uniquely attributable to non-methodological (e.g., season, water type, year) and methodological (e.g., sample type, volume).

According to variance partitioning analysis, the variance in the likelihood of detecting avian, canine, human, porcine, and ruminant markers that was jointly attributable to methodological and non-methodological factors was 13%, 6%, 12%, 1%, and 18%, respectively (Fig. 1; Table S1), indicating that, except for porcine, a large percentage of variance could be could not be attributed to either methodological or non-methodological factors. For avian markers, the variance in the likelihood of detection that was uniquely attributable to non-methodological factors (24%) was much larger than that attributable to methodological factors (<1%). Similar results were observed for canine (non-methodological: 10%; methodological: <1%), human (non-methodological: 38%; methodological: <1%), porcine (non-methodological: 31%; methodological: 2%), and ruminant markers (non-methodological: 22%; methodological: 2%; Fig. 1; Table S1).

### Top-ranked factors associated with detection of microbial source tracking markers according to conditional random forest

To compare the relative strength of the association between methodological and non-methodological factors after removing waterway and site-specific signals, conditional random forest analysis was performed. According to conditional forest, the three top-ranked factors most strongly associated with avian marker detection were, in order, year, sample volume, and season (Table 2). The top three factors associated with canine and human marker detection were season, sample volume, and water type (fine-scale), and state, season, and water type (fine-scale), respectively (Table 2). The top factors associated with porcine detection were sample volume, year, and season, and ruminant marker detection were the use of CowM3 as a molecular target, state, and year (Table 2).

**TABLE 2:** Variable importance rankings for factors included in conditional forests built using methodological and non-methodological features to understand the relative impact of methodological differences on observed water quality^*h*^

	Conditional variable importance				
	Avian^*a*^	Canine^*b*^	Human^*c*^	Porcine^*d*^	Ruminant^*e*^
Freshwater status^*f*^	2.98 * 10^−6^	0.00	7.95 * 10^−6^	−1.37 * 10^−5^	2.58 * 10^−7^
Sample type			2.24 * 10^−4^		6.30 * 10^−5^
Sample volume	3.84 * 10^−3^	1.96 * 10^−3^	3.22 * 10^−4^	1.77 * 10^−2^	1.18 * 10^−3^
Season	2.62 * 10^−3^	4.08 * 10^−3^	5.20 * 10^−3^	8.39 * 10^−3^	2.70 * 10^−3^
State	1.18 * 10^−3^	7.63 * 10^−5^	8.50 * 10^−3^	2.81 * 10^−3^	3.21 * 10^−3^
Water type ^*g*^					
Fine-scale	2.63 * 10^−3^	8.00 * 10^−5^	4.71 * 10^−3^	6.13 * 10^−5^	1.29 * 10^−4^
Mid-scale	1.86 * 10^−3^	1.88 * 10^−5^	3.31 * 10^−3^	4.71 * 10^−5^	1.82 * 10^−4^
Coarse-scale	3.19 * 10^−6^	0.00	8.58 * 10^−6^	0.00	0.00
General	2.25 * 10^−3^	9.31 * 10^−6^	1.30 * 10^−3^	−6.06 * 10^−5^	6.36 * 10^−6^
Year	1.75 * 10^−2^	4.82 * 10^−5^	1.03 * 10^−3^	9.48 * 10^−3^	2.90 * 10^−3^

^*a*^ Three binary dummy variables were also included for if a given genetic marker target was used to detect avian fecal contamination: GFD (variable importance [VI] = 8.94 * 10^−4^), goose (VI = 0.00), and LEESeagull (VI = 8.49 * 10^−4^).

^*b*^ Two binary dummy variables were also included for if a given genetic marker target was used to detect canine fecal contamination: DG37 (VI = 7.24 * 10^−6^) and dogBact (VI = 3.61 * 10^−5^).

^*c*^ Four binary dummy variables were also included for if a given genetic marker target was used to detect canine fecal contamination: HUMM2 (VI = 2.62 * 10^−4^), *B. theta* (VI = 1.30 * 10^−6^), HF183 (VI = 2.80 * 10^−4^), and MNIF (6.97 * 10^−4^).

^*d*^ Only one genetic marker target was used for detection of porcine fecal contamination by the studies included in the data set reported here.

^*e*^ Five binary dummy variables were also included for if a given genetic marker target was used to detect canine fecal contamination: BacR (VI = 5.07 * 10^−5^), CF128 (VI = 6.43 * 10^−5^), CF193 (VI = 2.17 * 10^−5^), CowM3 (1.48 * 10^−2^), and Rum2Bac (VI = 1.13 * 10^−5^).

^*f*^ If the water sample represents freshwater or brackish/saltwater.

^*g*^ Four levels of water type were considered in this study.

^*h*^ Prior to training the forest, separate generalized linear random effects models were fit to model likelihood of detecting each host-specific fecal marker using random effects of site nested in waterway; the dependent variable in the conditional forests was the residuals from these regression models.

Conditional forest analysis was used to identify the regional scheme that best reflects variability in the likelihood of detecting each MST marker; this is important for the development of guidance for managing fecal contamination risks at regional scales. Water type was also included as a covariate to help determine if separate guidance was needed for each water type in each region. After accounting for methodological, waterway, and site-specific signals, the three top-ranked factors for avian markers were water type (fine-scale), water type (mid-scale), and water type (general) (Table 3). The top-ranked factors associated with the likelihood of canine marker detection were the hydrological region, USDA region, and interstate climate region. The top-ranked factors associated with the likelihood of human marker detection were water type (fine-scale), water type (mid-scale), and water type (general). The top-ranked factors associated with the likelihood of ruminant marker detection were state, agricultural region scheme 3, and hydrological region (Table 3).

**TABLE 3:** Variable importance rankings for factors included in conditional forests that were built to determine if region^*a*^ or water type^*b*^ were associated more strongly with each microbial target after accounting for other confounding factors, and which regional scheme was most strongly associated with each host-specific fecal source tracking marker^*d*^

	Conditional variable importance			
	Host-specific fecal source tracking markers			
	Avian	Canine	Human	Ruminant
Agricultural region				
Scheme 1	3.18 * 10^−3^	2.09 * 10^−4^	1.63 * 10^−3^	5.65 * 10^−4^
Scheme 2	8.85 * 10^−4^	9.44 * 10^−5^	2.33 * 10^−3^	3.74 * 10^−4^
Scheme 3	6.42 * 10^−4^	2.81 * 10^−5^	5.41 * 10^−3^	7.19 * 10^−3^
Scheme 4	8.72 * 10^−4^	4.43 * 10^−5^	1.92 * 10^−3^	3.11 * 10^−4^
Biome				
Census region	2.12 * 10^−2^	2.12 * 10^−3^	5.19 * 10^−5^	5.56 * 10^−4^
Climate region	2.53 * 10^−2^	2.53 * 10^−3^	2.08 * 10^−4^	1.02 * 10^−3^
Ecoregion				
Level I	6.89 * 10^−4^	8.89 * 10^−6^	5.62 * 10^−4^	5.38 * 10^−4^
Level II	2.68 * 10^−3^	4.20 * 10^−5^	1.37 * 10^−3^	7.86 * 10^−4^
EPA region	2.21 * 10^−2^	2.22 * 10^−3^	2.56 * 10^−5^	4.43 * 10^−4^
Freshwater status	6.70 * 10^−3^	6.70 * 10^−4^	−1.69 * 10^−6^	0.00
Habitat type				
Aquatic	2.83 * 10^−4^	1.49 * 10^−6^	9.91 * 10^−4^	4.17 * 10^−4^
Terrestrial	5.82 * 10^−3^	−1.18 * 10^−6^	1.42 * 10^−3^	3.04 * 10^−3^
Hydrological region	5.18 * 10^−3^	5.18 * 10^−3^	2.29 * 10^−5^	4.29 * 10^−3^
State	3.11 * 10^−3^	1.41 * 10^−5^	1.30 * 10^−2^	9.35 * 10^−3^
USDA region	3.11 * 10^−2^	3.11 * 10^−3^	9.75 * 10^−5^	1.49 * 10^−3^
Water type				
Fine-scale	4.26 * 10^−2^	1.03 * 10^−3^	3.84 * 10^−2^	3.71 * 10^−4^
Mid-scale	3.34 * 10^−2^	7.34 * 10^−4^	2.83 * 10^−2^	1.95 * 10^−4^
Coarse-scale	2.45 * 10^−3^	0.00	2.44 * 10^−4^	2.18 * 10^−4^
General	2.67 * 10^−2^	1.07 * 10^−4^	1.76 * 10^−2^	–^*c*^

^*a*^ Using GPS coordinates and county, samples were classified into regions using 14 different regional schemes.

^*b*^ Freshwater status as well as four levels of water type.

^*c*^ – indicates that the values for the given feature were constant for all samples with data for the given target. As such, that feature was not included in the feature set for forest development.

^*d*^ Prior to training the forest, separate generalized linear random effects models were fit to model the likelihood of detecting each pathogen using random effects for each methodological factor available for the given target; the dependent variable in the conditional forests was the residuals from these regression models.

## DISCUSSION

### Method standardization is needed to reduce variability in host-specific microbial source tracking data

The variance partition analysis results revealed that variance attributable to methodological (e.g., water volume tested) differences could not be disentangled from the variance attributable to non-methodological factors (e.g., region, waterway). Variations in methodologies among studies pose a challenge to comparing findings. This lack of comparability makes it difficult to determine and develop effective strategies aimed at mitigating public health risks associated with fecal contamination of waterways.

After accounting for waterway and site-specific signals, conditional forests indicated that method was strongly associated with MST marker detection. For example, sample volume was among the top-ranked factors impacting the likelihood of detection for three of the five host-specific MST markers. Previous work in surface and drinking water has found that water volume is associated with the recovery of foodborne pathogens (44–46), viruses (47), and coliphages (48). Interestingly, while increasing volumes of water tested have been shown to be positively associated with odds of detection of certain bacteria (44–46), the impact of volume on viruses and coliphages appears to be dependent on the water source (47, 48). Since the results presented here demonstrate that volume was associated with avian, canine, and porcine detection, a standardized sample volume is needed to ensure that studies accurately capture and compare quantities of MST markers.

In addition to volume, the genetic marker used for detection (methodological factor) impacted the ruminant MST marker detection, with CowM3 being the top-ranked factor impacting the likelihood of detection. A prior study revealed that the CowM3 genetic marker outperformed the CowM2 marker in fecal samples with the quantity of the CowM3 detected by qPCR being 32.6 times higher than that of CowM2 in the same DNA samples (49). However, an additional study of ruminant markers observed that the CowM2 qPCR method most accurately determined ruminant marker quantities when paired with either the BacR or Rum2Bac genetic marker (50). This discrepancy may stem from the fact that in the current study, all positive samples for CowM2 originated from a single study, which had an overall positive rate of 90.6% (87/96).

However, it is crucial to acknowledge that when accounting for methodological differences, particularly those related to genetic markers, previous work examining MST markers and other molecular methods have highlighted considerable variability within and between laboratories (16, 50–54). Variation in the detection/recovery of microbial targets between laboratories has been attributed to differences in the number of replicate samples processed, supplies, equipment, and the skill set of laboratory personnel (16, 50–54). Variability in host-specific MST marker results has also been attributed to inconsistent data analysis techniques across laboratories, including variations in determining assay limits and the criteria for considering a sample positive based on the number of replicates needed (52, 54). The current study, along with previous research, demonstrates significant variability from multiple sources, which affects the ability to make meaningful comparisons and draw valid conclusions across different studies. While much of the inter- and intra-laboratory inconsistencies are difficult to control for, standardization in methods and sample processing protocols can aid in minimizing variability.

### Detection of host-specific MST markers was impacted by water type and location

In both methodological and non-methodological conditional forests, water type was among the top-ranked factors for the likelihood of detecting host-specific MST markers. Fine-scale water type consistently ranked higher than mid-scale, coarse-scale, and general water type across all host-specific MST markers, irrespective of the type of conditional forest analysis conducted. This suggests that water type accounts for more variability in the likelihood of detecting MST markers than other variables considered, and that water type-specific guidance for managing fecal contamination risk in recreational and agricultural waterways may be appropriate. Although there was greater variability in the methods conditional forest, the ranking of scales remained consistent for all host-specific MST markers in the non-methodological conditional forest (fine-scale > mid-scale > general scale > coarse scale). This consistency in ranking implies that the detection of MST markers varies substantially between specific water types, and one-size-fits-all approaches for management, even for similar water types (e.g., streams vs rivers), may not be appropriate. The finding of water type impacting the likelihood of detection is not unexpected, considering the inherent heterogeneity of aquatic environments. For instance, a study conducted on well and spring water sites in Switzerland found that human-associated MST markers were more prevalent in springs despite the sites being geographically close and having similar land use to the wells (55). The observed variation in microbial communities between the two sampling sites was likely influenced by differences in hydrological characteristics, such as flow dynamics, nutrient levels, and sediment composition.

In addition to water type, the US state emerged as a top-ranked factor impacting the likelihood of human and ruminant MST marker detection while hydrological region was a top-ranked factor for canine and ruminant MST marker detection. Hydrological regions are distinct geographical areas characterized by specific patterns of water flow, including river basins, watersheds, and drainage systems, and are often used to understand water resource distribution and management. Environmental conditions, such as climate and land use, are expected to vary across different regional classifications. A considerable body of prior research has investigated MST marker detection in various waterways aiming to understand spatial classifications and has found that land use, particularly urban vs rural classifications, impacts the detection rate and host of MST markers present (5, 6, 23, 56, 57). Waterways, regardless of water type, have consistently shown a higher prevalence of human-specific MST markers in urban sources and non-human markers (e.g., ruminant, bovine) in rural sources (5, 6, 23, 56, 57). For instance, a study in a NY creak noted that ruminant markers were detected in 58% (7/12) of rural site samples and 22% (4/18) of urban site samples, while human markers were detected in 33% (4/12) of samples from rural sites and 72% (13/18) of samples from urban sites (5). Overall, the finding of water type and location as important predictors of MST marker detection suggests the potential for developing specific regional and water-type guidance to manage fecal contamination.

### Temporal factors ranked among the top influences on MST marker detection

While microbial water quality is strongly affected by local environmental factors, temporal factors (e.g., year, season) still emerged as being strongly associated with each host-specific target. Season was a top-ranked factor associated with the likelihood of canine, human, avian, and porcine MST marker detection, while year was a top-ranked factor associated with the likelihood of avian, porcine, and ruminant MST marker detection. Variability in MST marker prevalence over time is expected due to the complex interplay of environmental conditions (e.g., temperature, rainfall, sunlight) as well as human and animal activities that affect fecal contamination and persistence in waterways. The relationship between season and environmental factors that are associated with season on MST marker prevalence and concentration is well-documented (8, 23, 26, 58, 59). A study examining the prevalence of human, porcine, and bovine MST markers at five locations spanning diverse climatic regions ([i] the Negro River in Brazil, [ii] the Glafkos River in Greece, [iii] the Tisza River in Hungary, [iv] the Llobregat River in Spain, and [v] the Umeälven River in Sweden) observed seasonal variations in MST marker prevalence (8). However, seasonal differences in prevalence were found to be specific to both the host and the location, as anticipated due to the differing climates and animal behaviors among the distinct ecosystems of each of the five sampling sites (8). Furthermore, water temperature was a top predictor of HF183 in Virginia waterways by the Random Forest analysis, with concentrations of HF183 decreasing with increasing water temperature (23). Seasonal variability is well documented in climates where seasons are categorized into dry and rainy seasons, as opposed to four distinct seasons (8, 58, 59). For instance, in Costa Rica, concentrations of the human MST marker HF183 were found to increase during the rainy season, irrespective of water type (58). These findings underscore the intricate relationship between temporal factors, environmental dynamics, and MST marker prevalence, highlighting the need for comprehensive monitoring and management strategies tailored to local conditions and seasonal variations.

### Conclusion

The present study, which compiled a data set of various host-specific MST markers from various geographical regions and water types while encompassing diversity in techniques, highlights that differences in methods limit the ability to compare findings across studies. Understanding fecal contamination of waterways and how prevalence is influenced by environmental and temporal factors is already complex, without the added complications of methodological differences; thus, standardization in sampling and laboratory methods is needed. However, once methods were accounted for, results demonstrated that environmental as well as spatial and temporal factors impacted the likelihood of detecting host-specific MST markers highlighting that distinct environments and processes drive water quality. This research contributes data on the intricacies of host-specific fecal contamination in the aquatic environment and demonstrates the establishment of one-size-fits-all standards or management practices for water may not be applicable.

## MATERIALS AND METHODS

### Data sets

Data points were collected and processed following the protocol previously described in (46). Briefly, a data frame was assembled using MST marker data and water quality data collected from peer-reviewed publications, citizen science and government monitoring programs, and publicly accessible databases. For each data set collected and retained, each data point was characterized by fresh vs saltwater, water type (e.g., lake/reservoir, river, pond, canal), and waterway name (e.g., Cedar Creek, Polk Lake); if geo-referenced information was not available, data were obtained using Google Earth and Google Maps. Furthermore, each data point was assigned to one of four unique nested classifications of water type, as previously described in (46). The broadest classification included three categories: groundwater, manure or wastewater, and surface water. Within these broad categories, there were 10 coarse-scale water types (e.g., lakes/ponds/reservoirs, runoff, and streams/rivers), 20 mid-scale water types (e.g., lakes or reservoirs, ponds, tidal streams), and 27 fine-scale water types (e.g., channelized streams, non-tidal streams, springs, salt ponds). If GPS coordinates and/or water type could not be determined for a data point, samples were excluded. Any relevant metadata (e.g., weather, physicochemical water quality) were also retained. Key methodological details (e.g., water testing volume, collection method) were sourced from previously published literature, accessible through web portals, or provided by data set owners via personal correspondence. For samples where there was insufficient methodological metadata, samples were excluded.

### Regions

Each data point was classified into regions using various regional schemes based on the GPS coordinates and county of samples. Numerous regional schemes were used to determine if there was a regional scheme that best accounted for variance. Fourteen separate regional schemes were considered, including schemes based on ecoregion, aquatic and terrestrial habitat type, biome, hydrologic region (USGS HUC2 unit codes), interstate climate region, regional schemes used by US federal agencies (i.e., Census Regions and US Environmental Protection Agency), and four schemes based on agricultural practices and/or output (USDA – Farm Resource Regions, USDA – Farm Production Regions, USDA-National Agricultural Statistics Service Regions, Human Geography Agricultural Regions [http://www2.harpercollege.edu/mhealy/geg100/notes/notes12x_files/f3_15_pg165.jpg]). For some regional schemes, all data points in a state were assigned the same region based on the region that covered a majority of the state. For the remaining schemes, region was extracted from publicly available shapefiles and could vary both within and between states.

### Statistical analysis

Data for each MST marker was summarized as prevalence (presence-absence). All analyses and data visualizations were performed in R version 4.0.3 (R Foundation for Statistical Computing, Vienna, Austria). More information regarding R packages and model specifications needed for each analysis can be found in Table 3 of reference (46).

### Attributable variance

To quantify the variance that is uniquely and jointly attributable to methodological and non-methodological factors, variance partitioning analysis was employed using the varpart function in the vegan package in R (60). Uniquely attributable variance refers to the portion of variance specifically attributed to one independent variable (i.e., methodological or non-methodological factors), while jointly attributable variance represents the variance that results from the combined effects of methodological and non-methodological factors. Four sets of variance partitioning analysis were performed to compare attributable variance: (i) state and region, site-specific (waterway, site, water type, freshwater status), temporal (season, year), and methodological factors (e.g., sample type, filter type, genetic marker used for confirmation or detection), (ii) waterway (waterway and sampling site), water type (water type and freshwater status), temporal and methodological factors, (iii) methodological and all non-methodological factors, and (iv) sampling site, methodological factors, and all other non-methodological factors.

### Conditional forest analysis

Conditional forest analysis, which can account for correlated factors, was used to characterize hierarchical associations between spatial, temporal, and methodological factors to better understand if and when methodological differences affected observed microbial water quality. Prior to training the forest, a generalized linear model (for binary outcomes) or general linear model (for continuous outcomes) was fit using the lme4 package (61) and random effects for each methodological factor available for the given target. If the model had a singular fit or failed to converge, it was re-parameterized (e.g., one methodological variable dropped from the model, random effect shifted to fixed effect). For each forest, unbiased conditional forest analysis was implemented using the moreparty package (62). Conditional variable importance was calculated to identify features in each forest that were most strongly associated with a given target. Due to the limited number of porcine samples and the lack of variability, conducting random forest analysis on porcine samples for region and water type was not feasible.

## Data Availability

Table 1 provides an overview of the data sources utilized in this study, acknowledging the original study authors and data owners. The data sets are available at: https://github.com/wellerd2/Weller-et-al-2024-AEM-Datasets/tree/main, or by reaching out to the study authors. Confidential data including GPS coordinates, site names, and waterway names were excluded due to privacy concerns related to commercial agricultural farms. However, this data can be made available from the original study authors upon request.

## SUPPLEMENTAL MATERIAL

Table S1 spectrum.01972-24-s0001.docx Variance partitioning analysis results for host-specific fecal source tracking markers.

ASM does not own the copyrights to Supplemental Material that may be linked to, or accessed through, an article. The authors have granted ASM a non-exclusive, world-wide license to publish the Supplemental Material files. Please contact the corresponding author directly for reuse.
