# Supplementary material for: The probability of detecting host-specific microbial source tracking markers in surface waters was strongly associated with method and season
Source: Microbiol Spectr. 2024 Dec 17;13(2):e01972-24. doi: 10.1128/spectrum.01972-24 (PMC11792506; doi:10.1128/spectrum.01972-24)
Supplement: Table S1 — Variance partitioning analysis results for host-specific fecal source tracking markers. [file spectrum.01972-24-s0001.docx]

**Supplemental Table 1: Variance partitioning analysis results for host-specific fecal source tracking markers.**

| Model Set | Matrix | Conditional Variance (Marginal Variance) ^a b^ | | | | |
| --- | --- | --- | --- | --- | --- | --- |
|  |  | Avian | Canine | Human | Porcine | Ruminant |
| One |  |  |  |  |  |  |
|  | Non-Methodological ^c^ | 0.24 (0.35) | 0.10 (0.16) *** | 0.38 (0.50) *** | 0.31 (0.40) *** | 0.22 (0.38) *** |
|  | Methodological^d^ | 0.01 (0.13) | < 0.01 (0.06) | < 0.01 (0.12) *** | 0.02 (0.01) *** | 0.02 (0.18) *** |
|  | Residual Variance | 0.64 | 0.84 | 0.50 | 0.58 | 0.60 |
| Two |  |  |  |  |  |  |
|  | Region ^e^ | < 0.01 (0.09) ** | < 0.01 (0.06) | 0.02 (0.28) *** | 0.00 (0.06) | < 0.01 (0.19) |
|  | Temporal ^f^ | < 0.01 (0.04) *** | 0.01 (0.04) *** | 0.01 (0.05) *** | 0.03 (0.11) *** | 0.02 (0.03) *** |
|  | Waterway (Set 1) ^g^ | 0.14 (0.22) *** | < 0.01 (0.04) | 0.12 (0.36) *** | 0.11 (0.17) *** | 0.05 (0.27) *** |
|  | Methodological ^d^ | 0.02 (0.13) *** | < 0.01 (0.05) | < 0.01 (0.12) ** | 0.01 (0.01) ** | 0.02 (0.18) *** |
|  | Residual Variance | 0.68 | 0.94 | 0.58 | 0.75 | 0.69 |
| Three |  |  |  |  |  |  |
|  | Waterway (Set 2) ^h^ | 0.01 (0.22) *** | < 0.01 (0.04) | 0.12 (0.36) *** | 0.11 (0.17) *** | 0.09 (0.27) *** |
|  | Temporal ^f^ | < 0.01 (0.04) *** | 0.01 (0.04) *** | 0.01 (0.05) *** | 0.03 (0.11) *** | 0.02 (0.03) *** |
|  | Water Type ^i^ | < 0.01 (0.07) | 0.00 (0.02) | < 0.01 (0.23) | 0.00 (0.02) | < 0.01 (0.04) |
|  | Methodological^d^ | 0.05 (0.13) *** | 0.01 (0.06) ** | 0.02 (0.12) *** | 0.01 (0.01) ** | 0.02 (0.18) *** |
|  | Residual Variance | 0.69 | 0.94 | 0.60 | 0.75 | 0.69 |

**^a^** The number of stars indicates the *P-value;* specifically, *** indicates *P* < 0.01, ** indicates *P* <0.01, and * indicates *P* <0.05.

^b^ Conditional variance represents the variance uniquely attributable to the given factor set (e.g., to methodological factors), while marginal variance represents the variance attributable to that factor set and to one or more other factor sets considered.

^c^ Includes all non-methodological factors, including waterway, sampling site, water type (fine, mid, coarse, and general), freshwater status, state, year, and season.

^d^ Includes all methodological factors, such as sample type, filter type, and if a given gene was used for confirmation or detection.

^e^ Matrix representing state and other “region” factors, such as USDA region and Census Region.

^f^ Matrix representing temporal factors, including year and season.

^g^ Matrix representing waterway that includes water type (fine, mid, coarse, and general), freshwater status, waterway, and sampling site.

^h^ Matrix representing waterway that includes waterway and sampling site but not water type (fine, mid, coarse and general) and freshwater status.

^i^ Matrix representing water type that includes water type (fine, mid, coarse, and general), and freshwater status.
